# Intelligent Reflecting Surface-Assisted Secure Multi-Input Single-Output Cognitive Radio Transmission

**DOI:** 10.3390/s20123480

**Published:** 2020-06-19

**Authors:** Haitao Xiao, Limeng Dong, Wenjie Wang

**Affiliations:** 1 School of Information and Communication Engineering, Xi’an Jiaotong University, No.28, Xianning West Road, Xi’an 710049, China; hjf2020_1@hotmail.com (L.D.); wjwang_1971@hotmail.com (W.W.); 2 Graduate School of Information, Production and Systems, Waseda University, 2–7, Hibikino, Wakamatsu-ku, Kitakyushu, Fukuoka 808–0135, Japan

**Keywords:** physical layer security, cognitive radio, intelligent reflecting surface, MISO, alternating optimization

## Abstract

Intelligent reflecting surface (IRS) is a very promising technology for the development of beyond 5G or 6G wireless communications due to its low complexity, intelligence, and green energy-efficient properties. In this paper, we combined IRS with physical layer security (PLS) to solve the security issue of cognitive radio (CR) networks. Specifically, an IRS-assisted multi-input single-output (MISO) CR wiretap channel was studied. To maximize the secrecy rate of secondary users subject to a total power constraint (TPC) for the transmitter and interference power constraint (IPC) for a single antenna primary receiver (PR) in this channel, an alternating optimization (AO) algorithm is proposed to jointly optimize the transmit covariance R at transmitter and phase shift coefficient Q at IRS by fixing the other as constant. When Q is fixed, R is globally optimized by equivalently transforming the quasi-convex sub-problem to convex one. When R is fixed, bisection search in combination with minorization–maximization (MM) algorithm was applied to optimize Q from the non-convex fractional programming sub-problem. During each iteration of MM, another bisection search algorithm is proposed, which is able to find the global optimal closed-form solution of Q given the initial point from the previous iteration of MM. The convergence of the proposed algorithm is analyzed, and an extension of applying this algorithm to multi-antenna PR case is discussed. Simulations have shown that our proposed IRS-assisted design greatly enhances the secondary user’s secrecy rate compared to existing methods without IRS. Even when IPC is active, the secrecy rate returned by our algorithm increases with transmit power as if there is no IPC at all.

## 1. Introduction

Cognitive radio (CR) was proposed as one of the most promising technologies aiming to solve the spectrum scarcity issues. Using spectrum sensing and sharing technology, the spectrum usage can be greatly improved so that the contradiction between exponential growth of wireless users and spectrum scarcity issues can be effectively relieved; however, CR networks are facing lots of security threads, such as primary user emulation, spectrum sensing data falsification, jamming, and eavesdropping [1]. In these attacks, eavesdropping brings great security risks due to the broadcast nature of wireless channels and open system architectures with wireless users in the same frequency band. To solve this security issue, physical layer security (PLS) has emerged as a very valuable technology to deal with eavesdropping attacks in wireless systems. In this approach, the properties of wireless channels are being fully utilized and by using signal processing strategy, and the transmitted information can be completely “hidden” from eavesdropping [2], which offers significant opportunities for enhancing the secrecy performance of CR networks [3].

### 1.1. Related Work

Secrecy rate (capacity) is the key issue of guaranteeing the user’s secret communication in PLS, and how to maximize the user’s secrecy rate has drawn wide attentions in the past decade. The multi-antenna wiretap channel (WTC) model became a popular tool to study PLS, the secrecy capacity of multiple-input single-output (MISO) and multiple-input multiple-output (MIMO) WTC subject to total power constraint (TPC) was deeply analyzed in [4,5]. Later, the channel was extended to the CR setting where an extra interference power constraint (IPC) at primary receiver (PR) was considered, and several solutions were proposed to maximize the secondary user’s secrecy rate [6,7,8,9,10,11,12,13]. The secrecy capacity optimization problem of CR MISO WTC was studied in [6], and several optimal solutions as well as sub-optimal beamforming solutions were proposed to solve this problem. This channel was extended to a special case where imperfect channel state information (CSI) was known at transmitter [7]. Furthermore, artificial noise (AN) approach was later proposed to maximize the secrecy rate of this channel under full/imperfect CSI [8,9]. Based on the study of MISO case, the secrecy capacity of CR MIMO WTC was also studied in [10], and several optimal analytical solutions under special cases [10,11] as well as optimal [12] and sub-optimal AN-aided [13] numerical solutions under general case were proposed to maximize the secrecy rate.

These existing solutions (such as AN and projected beamforming) for enhancing secrecy rate have obvious drawbacks due to two aspects. Firstly, although AN could effectively increase the user’s secrecy performance (especially for reverse degraded WTC case), it consumes certain amount of power at transmitter so that the residual power for signaling has reduced. Hence, the potential of AN method for boosting the secrecy rate is very limited. Secondly, different from non-CR settings with only TPC, as IPC is becoming active, full power allocation is not optimal for information signaling so that the secrecy rate saturates, which happens mostly when PR is located very close to secondary receiver. If putting full power for signaling under this case, the interference to PR can be easily beyond the predefined threshold so that the quality of service (QoS) at PR could be seriously affected [10]. Although the existing project beamforming solutions can null out the interference at PR so that IPC can be ignored [6,10], it decreases the degree of freedom at transmitter significantly. If PR is close to the secondary receiver, the signals could also be canceled out at receiver so that the secrecy rate of secondary user becomes zero; therefore, it is necessary to find an effective solution to not only boost the secondary user’s secrecy rate but also guarantee the QoS at PR without sacrificing any power as well as degree of freedom at transmitter.

Recently, intelligent reflecting surface (IRS), which is also called re-configurable intelligent surface, has been proposed and it has drawn wide attention for its applications in wireless communications. IRS is a software-controlled metasurface consisting of large numbers of passive reflecting elements. These elements induce certain phase shifts for the incident electromagnetic signal wave and reflect the signals with very low power consumption. It is confirmed by academia that IRS could play a key role in developing massive MIMO 2.0 for future’s beyond 5G or even 6G communications [14,15]. Since IRS is an intelligent metasurface, it has great benefits compared to the traditional reflecting surface, relaying systems and backscatter communications [15,16,17]. These significant advantages make IRS as a green energy-efficient technique and can be applied to various communication models such as multi-cell, massive device-to-device (D2D), wireless information and power transfer, and PLS [18]. The research of IRS was applied in to cognitive radio system and solutions ware discussed in [19,20,21,22]. In [19], an IRS-assisted multi-user cognitive radio (CR) channel model was formulated, and an algorithm was proposed to jointly optimize the beamforming vector at transmitter and continuous phase shift coefficients at IRS. Simulation results showed that the system sum rate is dramatically improved by proposed scheme compared to two baseline schemes. The authors of [20] introduce multiple IRSs to a downlink multiple-input single-output (MISO) CR system, where a single secondary user coexists with a primary network with multiple primary user receivers. The proposed algorithm jointly optimized the beamforming at the secondary user transmitter and the inflecting coefficient at each IRSn to maximize the achievable rate of the secondary user. Simulation results demonstrate that IRS can improve the achievable rate of secondary user. Furthermore, in [21], both bounded the CSI error model and statistical CSI error model for primary user (PU)-related channels in IRS aided CR systems were considered and the results indicate that the efficiency of the proposed algorithms and meaningful insights for the design of robust beamforming in IRS-aided CR systems. In addition, downlink multigroup multicast communication systems assisted by an IRS was consider in [22], where a huge increase of energy efficiency of the introduced IRS and the effectiveness in terms of the convergence and complexity were achieved.

All these contributions indicate that IRS greatly helps enhancing the transmission rate of users. Furthermore, IRS is currently also used to combine with PLS to overcome the eavesdropping attack issue in wireless networks [18]. By adjusting the phase shift coefficients, the reflected signal by IRS is not only added constructively with the non-reflected signal at the user, but also added destructively with the non-reflected signal at eavesdropper. As a consequence, the signal-to-noise (SNR) ratio is increased at the user and decreased at the eavesdropper so that higher secrecy rate can be achieved. Several latest research results about secure IRS-assisted MISO WTC were established in [23,24,25,26,27,28]. In [23,24], it was shown that IRS significantly improves the user’s secrecy rate compared with no-IRS case. A special case where no direct link between transmitter and receiver was considered in [25,26]. The multi-user MISO downlink wiretap channel was studied in [27], and also a special case where there is no direct link between transmitter and receiver was studied in [28]. All these results again indicate that IRS greatly boosts user’s secrecy rate rather than no-IRS case.

### 1.2. Contributions

Motivated by the aforementioned research results, we applied the IRS into the PLS of the CR issue in this paper, and focus on enhancing the secondary user’s secrecy rate. Compared to the other no-IRS algorithms, the main reasons for choosing IRS to enhance the secrecy performance in our work are due to these key aspects: Firstly, since the signal is destructively added to the eavesdropper with the aid of IRS, it is also possible to make the signals destructively add to the PR, hence the secrecy rate could be boosted and also the QoS at PR is satisfied. Secondly, IRS is an energy-efficient passive reflector, it neither consumes any power itself, nor sacrifices any part of power at transmitter, and the degree of freedom for transmission also can be increased by deploying more reflecting elements on IRS. Therefore, IRS has significant advantages compared with conventional AN and project beamforming solution. Finally, PLS is of great importance on the development of secure 5G communications, our work of combing IRS with PLS brings new ideas and thoughts to solve the security issues for beyond 5G or 6G based CR networks.

Based on the aforementioned aspects, the main contributions of our paper are summarized as follows:

1. In this paper, we set up an IRS-assisted Gaussian CR MISO wiretap channel, and focus on maximizing the secrecy rate of secondary user by jointly optimizing the transmit covariance at transmitter as well as phase shift coefficient at IRS subject to IPC at a PR with single antenna in addition to TPC at secondary transmitter. To the best of our knowledge, IRS has never been applied into the study of secure CR issue. Our work makes up for the lack of research in this area.

2. The formulated secrecy rate optimization problem is a difficult non-convex problem; therefore, an iterative alternating optimization (AO) algorithm is proposed, which is based on optimizing the transmit covariance matrix for secondary transmitter and phase shift matrix for IRS by fixing the other as a constant in two sub-problems. When the phase shift coefficient is fixed, the global optimal solution of transmit covariance is obtained by equivalently transforming the quasi-convex sub-optimization problem to a convex one. When the transmit covariance is fixed, the sub-optimal solution of phase shift coefficent is optimized in the non-convex fractional programming problem by using bisection search algorithm in combination with minorization–maximization (MM) algorithm. Due to the non-convexity of the constraints, it is still difficult to optimize the phase shift coefficient during each iteration of MM algorithm; therefore, another bisection search algorithm is proposed which is able to find the global optimal closed-form solutions of phase shift coefficient given fixed initial point from the previous iteration of MM algorithm.

3. The convergence of the AO algorithm is analyzed in detail by proposing a series of propositions, from which the solution returned by the AO algorithm is guaranteed to converge to a limit point. It is shown that the proposed algorithm also applies for multi-antenna PR case. Simulation results indicate that our proposed IRS-assisted design greatly boosts the secondary user’s secrecy rate. More importantly, even when PR and the secondary receiver are located in the same direction, full power allocation at secondary transmitter is still optimal so that the secrecy rate returned by our algorithm is still increasing with transmit power. This is significantly different from the no-IRS case in which the secrecy rate saturates gradually as transmit power is increasing since IPC is becoming active and full power allocation is not optimal signaling strategy.

The rest of the paper is organized as follows: Section 2 describes the channel model and formulate the optimization problem. In Section 3, the AO algorithm is proposed to jointly maximize the transmit covariance and phase shift coefficient. Simulations were carried out to evaluate the performance and convergence of the proposed algorithm, with results shown in Section 4. Finally, Section 5 concludes the paper.

*Notations*: bold lower-case letters (a) and capitals (A) denote vectors and matrices respectively; $A^{T}$, $A^{*}$, and $A^{H}$ denote transpose, conjugate, and Hermitian conjugate of A, respectively; A≥0 means positive semi-definite; E{·} is statistical expectation, $\lambda_{i}\left(A\right)$ denotes eigenvalues of A, which are in decreasing order unless indicated otherwise, i.e., $\lambda_{1}\geq \lambda_{2}\geq \lambda_{3}...$; |A| and tr(A) are determinant and trace of A; I is an identity matrix of appropriate size; $C^{M\times N}$ and $R^{M\times N}$ denotes the space of M×N matrix with complex-valued elements and real-valued elements, respectively; arg(a) denotes the phase of each entry of a; diag(a) is to transform the vector a to a diagonal matrix in which all diagonal entries are in a; Re{a} denotes the real element of a. A(i,j) denotes the entry in the *i*-th row and *j*-th column of A; ⊙ denotes Hadamard product; N(A) denotes the null space of A and R(A) denotes the range of A. rank(A) denotes the Rank of the A.

## 2. Channel Model and Problem Formulation

**Figure 1:**
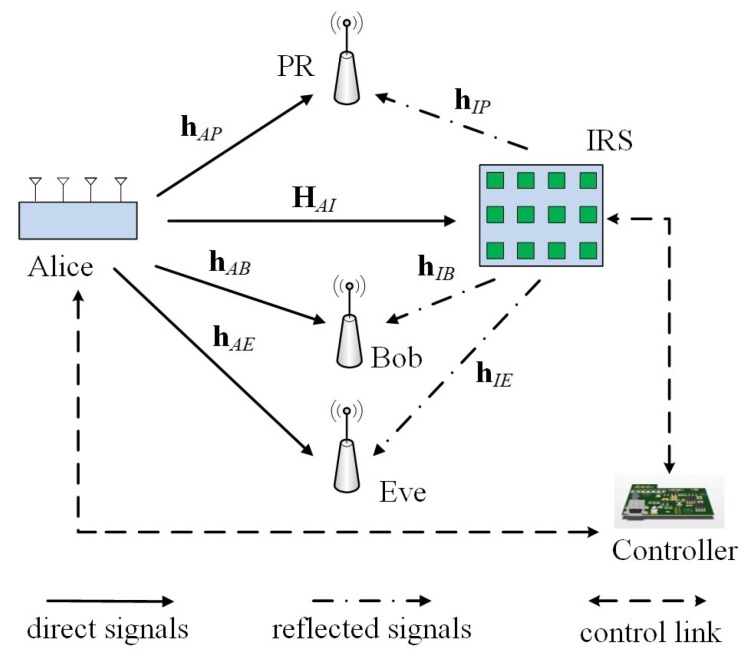
A block diagram of intelligent reflecting surface (IRS)-assisted Gaussian cognitive radio (CR) multi-input single-output (MISO) wiretap channel.

Let us consider an IRS-assisted MISO wiretap channel model shown as Figure 1 secondary transmitter Alice, a secondary receiver Bob, an eavesdropper Eve, and a PR and an IRS are included. The antenna number deployed at Alice and the number of reflecting elements deployed on IRS are m and n, respectively, and Bob, Eve, and PR are all equipped with a single antenna. The task for IRS in this model is to help Bob to improve its secrecy performance on the condition that the QoS at PR is satisfied. Therefore, IRS adjusts the phase shift coefficient for the reflecting elements by its controller, and reflect the broadcasted information signals from Alice passively to Bob, Eve, and PR (without generating any extra noise) so as to constructively add to the non-reflected signal from Alice–Bob link and destructively add with the non-reflected signal from Alice–Eve. Meanwhile, the reflected signal also destructively add with the non-reflected one from Alice–PR link so that the interference to PR does not exceed the pre-defined threshold. Our goal is to maximize the achievable secrecy rate of Bob by jointly optimizing the transmit covariance at Alice and phase shift coefficient at IRS.

Based on this setting, the received signals at Bob and Eve are expressed as

(1) $$\begin{array}{c} y_{B}=h_{AB}x+h_{IB}QH_{AI}x+\xi_{B} \end{array}$$

(2) $$\begin{array}{c} y_{E}=h_{AE}x+h_{IE}QH_{AI}x+\xi_{E} \end{array}$$

respectively, where $x\in C^{M\times 1}$ denotes the information signals, $h_{AB}\in C^{1\times M}$, $h_{AE}\in C^{1\times M}$, $h_{IB}\in C^{1\times N}$, $h_{IE}\in C^{1\times N}$, and $H_{AI}\in C^{N\times M}$ are the channel vectors (matrix) representing the direct link of Alice–Bob, Alice–Eve, IRS–Bob, IRS–Eve, and Alice–IRS respectively (direct link means the link for direct communication between transmitter and receiver, without hopping or relaying), $\xi_{B}$, and $\xi_{E}$ represent complex noise at Bob and Eve respectively, $Q=diag({\left[q_{1},q_{2},\ldots ,q_{n}\right]}^{T}$ ), $q_{i}$ = $e^{j\theta_{i}}$ is the diagonal phase shift matrix for IRS, $\theta_{i}$ is the phase shift coefficient at reflecting element *i*. In addition, the received signals at PR are also expressed as

(3) $$\begin{array}{c} y_{P}=h_{AP}x+h_{IP}QH_{AI}x+\xi_{P} \end{array}$$

where $h_{AP}\in C^{1\times M}$,$h_{IP}\in C^{1\times N}$, are the channel vectors representing the direct link of Alice–PR and IRS–PR respectively, $\xi_{P}$ represent complex noise at PR. Without loss of generality, it is assumed that all the noise $\xi_{B}$, $\xi_{E}$ and $\xi_{P}$ are distributed as CN(0,1) throughout the paper. We consider that full CSI is available to Alice and IRS, which can be achieved by modern adaptive system design, where channel is estimated at Bob and PR, and then sent back to the Alice and IRS via a feedback link; when Eve is just another user accessed in the system, they also share their CSI with Alice and IRS (In practice, Alice and IRS may not be able to have perfect CSI of Alice, Eve, and IRS–Eve link if Eve is a hidden unidentified user, and IRS also may requires extra equipment to obtain the CSI of IRS–Bob and IRS–PR link. The results in this paper serve as a theoretical performance upper bound for the considered real system.). Meanwhile, Alice and IRS could also exchange the CSI between each other via the control link. In this paper, we only consider using the CSI (channel state information), and all the optimization are based on this setting, thus the presented solution does not consider the effects of multi-path in the non-line-of-sight(NLOS) scenario. We will consider these in the future.

Based on Equations (1)–(3), the secrecy rate maximization problem of this channel model is expressed as

$$\begin{array}{cc} P1: & \max_{R,Q}\;C_{s}\left(R,Q\right)\\ s.t. & \;\;R\geq 0,tr\left(R\right)\leq P_{T},\\ & \left(h_{IP}QH_{AI}+h_{AP}\right)R{\left(h_{IP}QH_{AI}+h_{AP}\right)}^{H}\leq P_{I},\\ & |q_{i}|=1,i=1,2,\ldots ,n. \end{array}$$

where

$$\begin{array}{c} C_{s}\left(R,Q\right)=log_{2}\left(\frac{1+\left(h_{IB}QH_{AI}+h_{AB}\right)R{\left(h_{IB}QH_{AI}+h_{AB}\right)}^{H}}{1+\left(h_{IE}QH_{AI}+h_{AE}\right)R{\left(h_{IE}QH_{AI}+h_{AE}\right)}^{H}}\right) \end{array}$$

and where $R=E\{xx^{H}\}$ denotes the transmit covariance at Alice, $P_{T}$ denotes total transmit power budget for Alice and $tr\left(R\right)\leq P_{T}$ is the TPC, $P_{I}$ denotes the maximum interference power budget for PR and $\left(h_{IP}QH_{AI}+h_{AP}\right)R{\left(h_{IP}QH_{AI}+h_{AP}\right)}^{H}\leq P_{I}$ is the IPC, the unit modulus constraint $|q_{i}|=1$ ensures that each reflecting element in IRS does not change any amplitude of the signals.

Before solving this problem, a few remarks are in order.

**Remark** **1.** *Note that in P1, we maximize the secrecy rate by optimizing the general covariance matrix R instead of transmit beamforming vector at Alice. The reason is that since extra IPC is considered here and also the secrecy rate is related to both R and Q; therefore, it is not known whether beamforming is still the optimal signaling strategy in this scenario. In fact, we will later propose an AO algorithm to optimize R by fixing Q, and prove that beamforming is still the optimal solution (i.e., the rank of optimal R is still 1).*

**Remark** **2.** *Different from the MISO CR secrecy capacity optimization problem without IRS in [6], the formulated optimization problem P1 is a new complicate non-convex optimization problem with two variables. There is no optimal (or sub-optimal) closed-form or numerical solutions for this problem currently. Although some numerical solutions are established for the IRS-assisted MISO WTC case in which only TPC at Alice is considered [23,24,25,26,27,28], they all fail to the CR setting when extra IPC at PR is considered. This is because with IPC, full power allocation for signaling at Alice is not always optimal unless IPC can be ignored as an inactive constraint.*

**Remark** **3.** *The key difficulty in solving P1 is how to optimize Q subject to the unit modulus constraint $|q_{i}|=1$, since this is a unique non-convex constraint and cannot be approximated to convex one by using some existing methods such as Taylor series expansion [13]. Q is also not a Hermitian matrix, thereby significantly increasing the difficulty of solving this problem.*

## 3. Alternation Optimization Algorithm

Since P1 is a complicate non-convex optimization problem with two variables, it is difficult to directly optimize R and Q simultaneously. We propose an iterative AO algorithm to solve the secrecy rate maximization problem P1 via solving the two sub-problems alternatively: maximizing R when Q is given and maximizing Q when R is given. As sufficiently enough iterations are reached, the corresponding results returned by the AO algorithm are guaranteed to local convergence.

### 3.1. Optimizing **R** Given **Q**

In this subsection, we focus on solving the sub-problem of maximizing R given Q. For simplicity, let

$$\begin{array}{c} h_{n}=h_{In}QH_{AI}+h_{An},n\in \left\{B,E,P\right\} \end{array}$$

Then given fixed Q, the sub-problem of optimizing R can be expressed as P2:

$$\begin{array}{cc} P2: & \max_{R}\frac{1+h_{B}Rh_{B}^{H}}{1+h_{E}Rh_{E}^{H}}\\ s.t. & \;\;R\geq 0,tr\left(R\right)\leq P_{T},h_{P}Rh_{P}^{H}\leq P_{I}, \end{array}$$

where the log function is ignored here due to its monotonicity. Since Q is fixed, $h_{B}$, $h_{E}$, and $h_{P}$ are all fixed vectors so that all the constraints for R are convex constraints. P2 is a quasi-convex optimization problem, which can be globally optimized via numerical solutions. Note that since IPC is considered, the closed form optimal beamforming solution proposed in [4] is not always the optimal solution for this problem, unless IPC is an inactive constraint. To optimize R, we transformed P2 to the following problem P3 by introducing a variable *t*:

$$\begin{array}{cc} P3: & \max_{R,t}t+h_{B}\tilde{R}h_{B}^{H}\\ s.t. & \;\;\tilde{R}\geq 0,t\geq 0,tr\left(\tilde{R}\right)\leq tP_{T},h_{P}\tilde{R}h_{P}^{H}\leq tP_{I},h_{E}\tilde{R}h_{E}^{H}+t=1, \end{array}$$

where $t={\left(1+h_{E}Rh_{E}^{H}+1\right)}^{-1}$, $\tilde{R}=tR$. It can be proved that the optimal solution in P3 is the feasible solution in P2, and the optimal solution in P2 is also the feasible solution in P3, also note that the objective function in both P2 and P3 are equivalent; therefore, P2 and P3 are equivalent. Since P3 is a convex optimization problem with linear objective and linear constraints, we use the standard optimization software package CVX [29] to optimize P3. Once the optimal $\tilde{R}$ and *t* are obtained, the optimal solution of R for P2 can be obtained directly.

Note that when only TPC is considered, it is proved that beamforming is the optimal solution for transmitter [4]. In fact, when IPC is included in the problem, beamforming is also the optimal solution in P2, from which the following proposition holds.

**Proposition** **1.** *Consider $R_{opt}$ is the optimal solution for P2 given Q, then $rank(R_{opt})\leq 1$ holds.*

**Proof.**  See Appendix A.1. □

Proposition 1 indicates that although IPC is considered, beamforming is still the optimal signaling strategy even when IPC is active. This conclusion holds for any settings of $P_{T}$, $P_{I}$ as well as any number of antennas deployed at PR. Note that the closed-form optimal beamforming results illustrated in [4] only applies for inactive IPC case, the closed-form solutions when IPC is active is still an open problem.

### 3.2. Optimizing **Q** Given **R**

In this subsction, we focus on our attention on solving the sub-problem of maximizing Q given R. Firstly, when R is fixed, P1 can be relaxed as the following sub-problem P4:

(4) $$\begin{array}{c} P4:\max_{Q}\frac{1+h_{B}Rh_{B}^{H}}{1+h_{E}Rh_{E}^{H}}\;\;\;s.t.\;\;h_{P}Rh_{P}^{H}\leq P_{I}, \end{array}$$

Apparently, this is a difficult non-convex fractional programming optimization problem with non-convex objective as well as non-convex constraints, especially the unit modulus constraint of Equation (4); therefore, we propose a solution to optimize a local optimal Q for this problem, the key idea in our paper is of three steps. Firstly, we temporarily fix Equation (4), and make manipulations to the objective as well as IPC $h_{B}Rh_{B}^{H}\leq P_{I}$ so as to make P4 to be a more tractable problem (shown as P5 and P6). Secondly, we propose bisection search algorithm in combination with iterative MM algorithm to solve this tractable problem. Thirdly, to overcome the non-convex constraint of Equation (4) in each iteration of MM algorithm, we propose another bisection search algorithm to obtain the global optimal Q given the initial point returned from the previous iteration of MM algorithm.

Specifically, let

$$\begin{array}{c} s={\left[q_{1}^{*},q_{2}^{*},...,q_{n}^{*}\right]}^{T} \end{array}$$

then for m∈{B,E,P},

$$\begin{array}{c} 1+h_{m}Rh_{m}^{H}=1+h_{Im}QH_{AI}RH_{AI}^{H}Q^{H}h_{Im}^{H}+h_{Am}RH_{AI}^{H}Q^{H}h_{Im}^{H}+h_{Im}QH_{AI}Rh_{Am}^{H}+h_{Am}Rh_{Am}^{H} \end{array}$$

(5) $$\begin{array}{c} =1+h_{Am}Rh_{Am}^{H}+s^{H}\left\{diag\left(h_{Im}^{T}\right)H_{AI}R{\left[diag\left(h_{Im}^{T}\right)H_{AI}\right]}^{H}\right\}s+2Re\left\{s^{H}diag\left(h_{Im}^{T}\right)H_{AI}Rh_{Am}^{H}\right\} \end{array}$$

(6) $$\begin{array}{c} =1+{\bar{h}}_{Am}+s^{H}{\bar{H}}_{Im}s+2Re\left\{s^{H}{\tilde{h}}_{Im}\right\} \end{array}$$

where Equation (5) holds since $a^{H}Qb=s^{H}diag\left(a^{H}\right)b$ holds for any vectors a and b with proper sizes, and where

$$\begin{array}{c} {\bar{h}}_{Am}=h_{Am}Rh_{Am}^{H}+s^{H},\;{\tilde{h}}_{Im}=diag\left(h_{Im}^{T}\right)H_{AI}Rh_{Am}^{H}\\ {\bar{H}}_{Im}=diag\left(h_{Im}^{T}\right)H_{AI}R{\left[diag\left(h_{Im}^{T}\right)H_{AI}\right]}^{H} \end{array}$$

Therefore, problem P4 can be equivalently expressed as the following P5:

(7) $$\begin{array}{cc} P5: & \max_{s}\frac{1+{\bar{h}}_{AB}+s^{H}{\bar{H}}_{IB}s+2Re\left\{s^{H}{\tilde{h}}_{IB}\right\}}{1+{\bar{h}}_{AE}+s^{H}{\bar{H}}_{IE}s+2Re\left\{s^{H}{\tilde{h}}_{IE}\right\}}\\ s.t. & (4),\\ & {\bar{h}}_{AP}+s^{H}{\bar{H}}_{IP}s+2Re\left\{s^{H}{\tilde{h}}_{IP}\right\}\leq P_{I} \end{array}$$

It can be known that P5 is more tractable problem compared with P4, since both the numerator and denominator of the objective function as well as the IPC are quadratic convex function respect to s. To optimize s in P5, we add a non-negative parameter u>0, and transform P5 to the following P6:

$$\begin{array}{cc} P6: & \;\min_{s}\;f\left(s/u\right)=1+{\bar{h}}_{AE}-u\left(1+{\bar{h}}_{AB}\right)+s^{H}\left({\bar{H}}_{IE}-u{\bar{H}}_{IB}\right)s+2Re\left\{s^{H}\left({\tilde{h}}_{IE}-u{\tilde{h}}_{IB}\right)\right\}\\ s.t. & (4),(7), \end{array}$$

Let s(u) denote the optimal value of P6 given fixed *u*, according to the key idea of fractional programming [30], finding the optimal solution of P5 is equivalent to searching for the optimal *u* such that the value of objective function f(s(u)/u)=0. In this paper, we apply bisection search algorithm to solve P5 according to the monotonic property of f(s(u)/u) in *u*.

Since P6 is still a non-convex optimization problem due to Equation (4), it is difficult to directly optimize *s* given fixed *u* in each iteration of bisection search. Although the existing semi-definite relaxation method proposed in [19,20,21,22,23] could solve P6 by properly transforming the objective and constraints (including Equation (4)) to convex formula so that CVX can be directly applied to optimize *s* by dropping the rank constraints, it requires significant computational complexity especially when *m* and *n* are large. Moreover, extra Gaussian randomization approach is needed to recover the rank-1 solution if the results produced by CVX is not rank-1, thereby further increasing the computational complexity; therefore, we propose the MM algorithm to solve P6 given *u* in each iteration of bisection search, which has been widely used in the applications of wireless signal processing. The key idea of MM algorithm is to firstly approximate the original problem by formulating an approximately upper bound of the objective and constraints, and then iteratively compute the optimal value of this approximated problem by initializing a feasible starting point. If the bound is constructed properly, any limit point of the solutions returned by MM algorithm is guaranteed to local convergence for the original problem [31].

To find the surrogate function of f(s/u) in P6, we apply the following Lemma illustrated in [32]:

**Lemma** **1.** *Let X be an n×n Hermitian matrix, then for any point $\tilde{a}\in C^{n\times 1}$, $a^{H}Xa$ is upper bounded by*

$$\begin{array}{c} a^{H}Xa\leq a^{H}Ya-2Re\left\{a^{H}\left(Y-X\right)\tilde{a}\right\}+{\tilde{a}}^{H}\left(Y-X\right)\tilde{a},\;\;\;where\;\;Y=\lambda_{1}\left(X\right)I \end{array}$$

Using this lemma, the upper bound of f(s/u) can be obtained as

(8) $$\begin{array}{c} f\left(s/u\right)\leq c_{1}\left(u\right)+c_{2}\left(u\right)+2Re\left\{s^{H}\left[\left({\tilde{h}}_{IE}-u{\tilde{h}}_{IB}\right)-\left(\lambda_{1}\left({\bar{H}}_{IE}-u{\bar{H}}_{IB}\right)I-\left({\bar{H}}_{IE}-u{\bar{H}}_{IB}\right)\right)\tilde{s}\right]\right\}\\ =\tilde{f}\left(s/\left(u,\tilde{s}\right)\right) \end{array}$$

where $\bar{s}$ is the solution returned by MM algorithm in the previous iteration, and where $c_{1}\left(u\right)$ and $c_{2}\left(u\right)$ are constant terms if u is fixed, which are expressed as

(9) $$\begin{array}{c} c_{1}\left(u\right)=1+{\bar{h}}_{AE}-u\left(1+{\bar{h}}_{AB}\right)+s^{H}\lambda_{1}\left({\bar{H}}_{IE}-u{\bar{H}}_{IB}\right)Is\\ =1+{\bar{h}}_{AE}-u\left(1+{\bar{h}}_{AB}\right)+n\lambda_{1}\left({\bar{H}}_{IE}-u{\bar{H}}_{IB}\right) \end{array}$$

(10) $$\begin{array}{c} c_{2}\left(u\right)={\tilde{s}}^{H}\left[\lambda_{1}\left({\bar{H}}_{IE}-u{\bar{H}}_{IB}\right)I-\left({\bar{H}}_{IE}-u{\bar{H}}_{IB}\right)\right]\tilde{s}\\ =n\lambda_{1}\left({\bar{H}}_{IE}-u{\bar{H}}_{IB}\right)-{\tilde{s}}^{H}\left({\bar{H}}_{IE}-u{\bar{H}}_{IB}\right)\tilde{s} \end{array}$$

respectively. Equations (9) and (10) hold since ${\tilde{s}}^{H}\tilde{s}=s^{H}s=n$. It can be verified that $\tilde{f}\left(s/\left(u,\tilde{s}\right)\right)$ is continuous in s and $\tilde{s}$ given fixed *u*, and also $\tilde{f}\left(s/\left(u,\tilde{s}\right)\right)=\tilde{f}\left(\tilde{s}/u\right)$, $\nabla_{s}\tilde{f}\left(s/\left(u,\tilde{s}\right)\right){|}_{s=\tilde{s}}=\nabla_{s}f\left(s/u\right){|}_{s=\tilde{s}}$ hold; therefore, $\tilde{f}\left(s/\left(u,\tilde{s}\right)\right)$ is a surrogate function of f(s/u), and also a linear function respect to s. Similarly, Equation (7) can also be approximated to an upper bound:

(11) $$\begin{array}{c} g\left(s\right)={\bar{h}}_{AP}+s^{H}{\bar{H}}_{IP}s+2Re\left\{s^{H}{\tilde{h}}_{IP}\right\}\leq c_{3}+2Re\left\{s^{H}\left[{\tilde{h}}_{IP}-\left(\lambda_{1}\left({\bar{H}}_{IP}\right)I-{\bar{H}}_{IP}\right)\tilde{s}\right]\right\}\\ =c_{3}+(\tilde{g}\left(s/\tilde{s}\right)\leq P_{I} \end{array}$$

where

$$\begin{array}{c} c_{3}\left(u\right)={\bar{h}}_{AP}+2n\lambda_{1}\left({\bar{H}}_{IP}\right)-{\tilde{s}}^{H}{\bar{H}}_{IP}\tilde{s}\\ \tilde{g}\left(s/\tilde{s}\right)=2Re\left\{s^{H}\left[{\tilde{h}}_{IP}-\left(\lambda_{1}\left({\bar{H}}_{IP}\right)I-{\bar{H}}_{IP}\right)\tilde{s}\right]\right\} \end{array}$$

Then, P6 can be approximated to a new optimization problem P7:

(12) $$\begin{array}{c} P7:\;\min_{s}\;\tilde{f}\left(s/\left(u,\tilde{s}\right)\right)\;\;s.t.\left(4\right),\\ \tilde{g}\left(s/\tilde{s}\right)\leq P_{I}-c_{3}={\tilde{P}}_{I} \end{array}$$

Using the MM algorithm to solve P7 by setting feasible initial point $\tilde{s}$, the output result is guaranteed to be a local optimal point of P6 (which will be proved later).

During each iteration of theMM algorithm, P7 is still difficult to solve due to Equation (4). In the following, we propose another bisection search algorithm to obtain a closed-form global optimal solution of P7 given initial point $\tilde{s}$. Specifically, we firstly write the Lagrangian of P7 as

$$\begin{array}{c} L\left(s,\mu ,\upsilon_{i}\right)=\tilde{f}\left(s/\left(u,\tilde{s}\right)\right)+\mu \left(\tilde{g}\left(s/\tilde{s}\right)-{\tilde{P}}_{I}\right)+\sum_{i=1}^{n}\upsilon_{i}\left(\right|q_{i}\left|-1\right), \end{array}$$

where μ and $\upsilon_{i}$ denote the Lagrange multipliers responsible for the constraints of Equations (12) and (4) respectively. Thus, the Karush Kuhn–Tucker (KKT) conditions of P7 expressed as

(13) $$\begin{array}{c} \nabla_{s}\tilde{f}\left(s/\left(u,\tilde{s}\right)\right)+\mu \nabla_{s}\tilde{g}\left(s/\tilde{s}\right)+\sum_{i=1}^{n}\upsilon_{i}\nabla_{s}\left|q_{i}\right|=0, \end{array}$$

(14) $$\begin{array}{c} \mu \left(\tilde{g}\left(s/\tilde{s}\right)\right)-{\tilde{P}}_{I})=0,\;\;\upsilon_{i}\left(\right|q_{i}\left|-1\right)=0, \end{array}$$

(15) $$\begin{array}{c} \tilde{g}\left(s/\tilde{s}\right))\leq {\tilde{P}}_{I},\;\;\left|q_{i}\right|=1, \end{array}$$

(16) $$\begin{array}{c} \mu \geq 0,\;\;\upsilon_{i}\geq 0,i=1,2,...,n, \end{array}$$

where Equation (13) is the stationary condition, Equation (14) contains the complementary slackness conditions, Equation (15) contains the primal feasibility conditions, and Equation (16) contains the dual feasibility conditions. According to [33], although P7 is non-convex problem, its optimal solution as well as dual optimal solutions must satisfy the above KKT conditions. Consider s(μ) is the optimal solution of P7, where μ is the corresponding dual optimal Lagrange multiplier, then

(17) $$\begin{array}{c} \mu \left(\tilde{g}\left(s\left(\mu \right)/\tilde{s}\right)\right)-{\tilde{P}}_{I})=0. \end{array}$$

Our goal is to search for the primal optimal s(μ) and dual optimal μ satisfying Equation (17).

It is straightforward to know that the optimal dual variable μ can be either zero or positive. We consider the simple case μ=0, which means Equation (12) is not active so that $\tilde{g}\left(s\left(\mu \right)/\tilde{s}\right))<{\tilde{P}}_{I}$. Therefore, P7 can be equivalently reduced to the following problem P8:

(18) $$\begin{array}{c} P8:\;\max_{s}\;-\tilde{f}\left(s/\left(u,\tilde{s}\right)\right)\;\;s.t.\left(4\right). \end{array}$$

Obviously, it can be known that the objective function in P8 is maximized only when each entry in *s* has same angular with those in $\tilde{q}$ where

$$\begin{array}{c} \tilde{q}=[\left(\lambda_{1}\left({\bar{H}}_{IE}-u{\bar{H}}_{IB}\right)I-\left({\bar{H}}_{IE}-u{\bar{H}}_{IB}\right)\right]\tilde{s}-\left({\tilde{h}}_{IE}-u{\tilde{h}}_{IB}\right). \end{array}$$

Therefore, the closed-form optimal solution for maximizing P8 is

(19) $$\begin{array}{c} s\left(\mu =0\right)=e^{jarg(\tilde{q})}, \end{array}$$

and Equation (19) is also the global optimal solution of P7 if Equation (12) is inactive.

The next case is when μ>0, i.e., Equation (12) is active so that $\tilde{g}\left(s\left(\mu \right)/\tilde{s}\right))={\tilde{P}}_{I}$. It can be known that Equation (19) is no longer the optimal solution for P8 in this case. To obtain the optimal solution, we firstly give the following proposition.

**Proposition** **1.** The function $\tilde{g}\left(s\left(\mu \right)/\tilde{s}\right))$ is a monotonically decreasing function in μ, where s(μ) is the optimal solution of P7 with given μ.

**Proof.**  See Appendix A.2. □

With this proposition, we add the term $\mu \tilde{g}\left(s\left(\mu \right)/\tilde{s}\right))$ after the objective function in P7, and consider the following problem P9:

(20) $$\begin{array}{c} P9:\;\max_{s}\;-\tilde{f}\left(s/\left(u,\tilde{s}\right)\right)-\mu \tilde{g}\left(s\left(\mu \right)/\tilde{s}\right))\;\;s.t.\left(4\right). \end{array}$$

It can be known that given fixed μ>0, the objective in P9 is maximized only when each entry in s has same angular with those in $\tilde{q}-\mu \bar{q}$, where

$$\begin{array}{c} \bar{q}={\tilde{h}}_{IP}-\left(\lambda_{1}\left({\bar{H}}_{IP}\right)I-{\bar{H}}_{IP}\right)\tilde{s}. \end{array}$$

Therefore, the closed-form optimal solution for P9 is expressed as

(21) $$\begin{array}{c} s\left(\mu >0\right)=e^{jarg(\tilde{q}-\mu \bar{q})}, \end{array}$$

and this is also the global optimal solution of P7 if μ is found such that the complementary slackers condition of Equation (17) is satisfied (which will be proved later). Since $\tilde{g}\left(s\left(\mu \right)/\tilde{s}\right))$ is decreasing in μ, one can find the optimal dual variable μ satisfying Equation (17) using existing numerical algorithm. Based on this property, we propose the bisection search algorithm again to search for the optimal μ of P7 under the active Equation (12) case, which is concluded as Algorithm 1. In this algorithm, given initial $\mu_{u}$ and $\mu_{l}$, the total number of iterations $k_{\epsilon_{1}}$ need to achieve the target accuracy $\epsilon_{1}$ for bisection search is $k_{\epsilon_{1}}=log_{2}\left(\left(\mu_{u}-\mu_{l}\right)/\epsilon_{1}\right)$. The main computational complexity lies in computing the eigenvalue $\lambda_{1}\left({\bar{H}}_{IP}-u{\bar{H}}_{IB}\right)$, $\lambda_{1}\left({\bar{H}}_{IP}\right)$ and computing s(μ) via Equations (19) or (21). Therefore, if Equation (12) is inactive, the total computational complexity is $o(n^{3}+n^{2})$, and if Equation (12) is active, the total complexity is $o(n^{3}+k_{\epsilon_{1}}n^{2})$. In the following, we show that the results returned by Algorithm 1 is the global optimal solution of P7.

**Proposition** **2.** *If Equation (12) is active, the output μ and s(μ) returned by Algorithm 1 is the global optimal and dual optimal.*

**Proof.**  See Appendix A.3. □

**Algorithm 1** (*Bisection algorithm to solve P7 if Equation (12) is active)***Require:** Initialize $\mu_{l}$ and $\mu_{u}$, set $\epsilon_{1}>0$.  **repeat**         1. Set $\mu =(\mu_{l}+\mu_{u})/2$.         2. Compute s(μ) via (21).         3. If $\tilde{g}\left(s\left(\mu \right)/\tilde{s}\right))\geq \tilde{P_{I}}$, set $\mu_{l}=\mu$, otherwise set $\mu_{u}=\mu$.  **until**$|\mu_{u}-\mu_{l}|\leq \epsilon_{1}$.  4. Output s(μ) as the global optimal solution of P7.

With Algorithm 1 at hand, we conclude the MM algorithm for solving P6 given fixed *u* shown as Algorithm 2. We set the initial point $\tilde{s}={\left[1,1,...,1_{n}\right]}^{T}$, and solve s in P7 iteratively until the target accuracy $\epsilon_{2}$ for convergence is reached. The following proposition shows the local convergence of Algorithm 2.

**Proposition** **3.** *In Algorithm 2, the sequence of the objective $f(s_{k}/u),k=1,2,...$ is guaranteed to converge with k and the output s is the local optimal solution of P6*.

**Proof.**  See Appendix A.4. □

**Algorithm 2** (*(Minorization–maximization (MM) algorithm to solve P6)***Require:** Initialize starting point $s_{0}$, set $\epsilon_{2}>0$.  1. Set k=0, compute $f_{0}=f\left(s_{0}/u\right)$.  **repeat**        2. Set k=k+1,        3. Compute $s_{k}$ via (19), if $\tilde{g}\left(s_{k}/s_{k-1}\right)>{\tilde{P}}_{I}$, then compute $s_{k}$ via Algorithm 1.        4. Compute $f_{k}=\tilde{f}\left(s_{k}/\left(u,s_{k-1}\right)\right)$.        5. Set $s_{k}$ as new starting point.  **until**$|f_{k}-f_{k-1}\left|/\right|f_{k-1}|\leq \epsilon_{2}$.  6. Output s as the local optimal solution of P6.

With Algorithms 1 and 2, the residual work solves the fractional programming P5, which is based on the following key proposition.

**Proposition** **4.** *Consider s(u) is the local optimal solution returned by Algorithm 2 given fixed u, then $f(s_{k}/u)$ is monotonically decreasing in u.*

**Proof.**  See Appendix A.5. □

Based on this proposition, the bisection search algorithm in combination with MM algorithm to search for the optimal u such that $f(\hat{s}\left(u\right)/u)=0$ is concluded as Algorithm 3. Same with Algorithm 1, the total number of iterations $k_{\epsilon_{3}}$ for achieving the target accuracy $\epsilon_{3}$ is $k_{\epsilon_{3}}=log_{2}\left(\left(u_{u}-u_{l}\right)/\epsilon_{3}\right)$.
**Algorithm 3** (*Bisection algorithm to solve P5)***Require:** Initialize $u_{l}$ and $u_{u}$, set $\epsilon_{3}>0$.  **repeat**      1. Set $u=(u_{l}+u_{u})/2$.      2. Compute $\hat{s}\left(u\right)$ via Algorithm 2      3. If $f(\hat{s}\left(u\right)/u)\geq 0$, set $u_{l}=u$, otherwise set $u_{u}=u$.  **until**$|u_{u}-u_{l}|\leq \epsilon_{3}$.  4. Output $\hat{s}\left(u\right)\to Q$.

### 3.3. Overall AO Algorithm

Finally, we conclude the overall AO algorithm for solving P1 is as Algorithm 4. In this algorithm, we set the feasible starting point $Q_{0}=I$ and $R_{0}=P_{T}I/am$ where a>0 is a adjustable constant so as to guarantee that both TPC and IPC in P1 are satisfied. Denote the objective function in P5 as h(s), based on the relationship between original function f(s/u) and surrogate function $\tilde{f}\left(s/\left(u,\tilde{s}\right)\right)$ in MM algorithm, one obtains that

(22) $$\begin{array}{c} h\left(s\right)\leq \frac{1}{u_{opt}}=h\left(s\left(u_{op}\right)\right) \end{array}$$

where $s(u_{opt})$ is the solution of P5 satisfying $f(s\left(u_{opt}\right)/u_{opt})=0$ returned by Algorithm 3. This indicates that the objective value of h(s) is non-decreasing during each iteration of AO algorithm. Since R and Q are optimized alternatively, and also R is the global optimal solution given fixed Q in P2, the value of objective $C_{s}\left(R,Q\right)$ in P1 is non-decreasing, i.e.,

(23) $$\begin{array}{c} C_{s}\left(R_{1},Q_{1}\right)\leq C_{s}\left(R_{2},Q_{2}\right)\leq ...\leq C_{s}\left(R_{k},Q_{k}\right), \end{array}$$

Furthermore, R and Q are both bounded by the constraints illustrated in P1 receptively. By applying the Cauchy’s theorem [27], one obtains that a solution $R_{opt}$ and $Q_{opt}$ always exist such that

(24) $$\begin{array}{c} 0=\lim_{k\to \infty }\;\left\{C_{s}\left(R_{k},Q_{k}\right)-C_{s}\left(R_{opt},Q_{opt}\right)\right\}\\ \leq \lim_{k\to \infty }\;\left\{C_{s}\left(R_{k+1},Q_{k+1}\right)-C_{s}\left(R_{opt},Q_{opt}\right)\right\}=0. \end{array}$$

which means $C_{s}\left(R_{k},Q_{k}\right)$ must converge to a limit point $C_{s}\left(R_{opt},Q_{opt}\right)$ eventually. Our extensive numerical experiments have validate that the monotonic convergence process of the AO algorithm is guaranteed.
**Algorithm 4** (*(Alternating optimization (AO) algorithm of solving P1)***Require:** Starting point $R_{0}$ and $Q_{0}$, $\epsilon_{4}>0$.  1. Set k=0, Compute $C_{s}\left(R_{0},Q_{0}\right)$.  **repeat**(AO algorithm)        2. Set k=k+1.        3. Optimize global optimal $R_{k}$ given fixed $Q_{k-1}$ via CVX.        4. Optimize local optimal $Q_{k}$ given fixed $R_{k}$ via Algorithm 3.        5. Compute $C_{s}\left(R_{k},Q_{k}\right)$.  **until**$|C_{s}\left(R_{k},Q_{k}\right)-C_{s}\left(R_{k-1},Q_{k-1}\right)\left|/\right|C_{s}\left(R_{k-1},Q_{k-1}\right)|\leq \epsilon_{4}$.  7. output $R_{k}$, $Q_{k}$ as a limit point of P1.

### 3.4. An Extension to Multi-Antenna PR Case

In this subsection, we consider that PR illustrated in Figure 1 is equipped with multiple antennas, and shows that the proposed AO algorithm still can be applied to this case by properly making some manipulations to the IPC. Specifically, consider R is equipped with $N_{p}$ antennas, then denote $H_{AP}\in C^{N_{p}\times M}$ and $H_{IP}\in C^{N_{p}\times M}$ as the channel matrices representing the direct link of Alice–PR and IRS–PR respectively. Then, the corresponding IPC in P1 is replaced by $tr\left(H_{P}RH_{P}^{H}\right)\leq P_{I}$ where $H_{P}=H_{AP}+H_{IP}QH_{AI}$. Since the objective function s unchanged, given fixed Q, the optimal R also can be obtained by solving P3 in which the constraint $h_{P}\tilde{R}h_{P}^{H}\leq tP_{I}$ is replaced by $tr\left(H_{p}\tilde{R}H_{P}^{H}\right)\leq tP_{I}$.

To obtain the solution of Q given fixed R, the IPC can be further expressed as

(25) $$\begin{array}{cc} tr(H_{P}RH_{P}^{H}) & =tr(H_{AP}RH_{AP}^{H}+H_{IP}QH_{AI}RH_{AP}^{H}+H_{AP}RH_{AI}^{H}Q^{H}H_{IP}^{H}+H_{IP}QH_{AI}RH_{AI}^{H}Q^{H}H_{IP}^{H})\\ & =c+tr\left(QA_{1}\right)+tr\left(Q^{H}A_{1}^{H}\right)+tr\left(Q^{H}H_{IP}^{H}H_{IP}QA_{2}\right) \end{array}$$

(26) $$\begin{array}{cc} = & c+2Re\left\{a_{1}q^{*}\right\}+q^{*H}\left(\left(H_{IP}^{H}H_{IP}\right)⊙A_{2}^{T}\right)q\\ = & c+2Re\left\{q^{*H}a_{1}^{H}\right\}+q^{*H}{\left(\left(H_{IP}^{H}H_{IP}\right)⊙A_{2}^{T}\right)}^{*}q^{*} \end{array}$$

$$\begin{array}{cc} = & c+2Re\left\{s^{H}a_{1}^{H}\right\}+s^{H}{\left(\left(H_{IP}^{H}H_{IP}\right)⊙A_{2}^{T}\right)}^{*}s\leq P_{I} \end{array}$$

where

$$\begin{array}{c} c=tr\left(H_{AP}RH_{AP}^{H}\right),A_{1}=H_{AI}RH_{AP}^{H}H_{IP},\\ A_{2}=H_{AI}RH_{AI}^{H},q={\left[q_{1},q_{2},...,q_{n}\right]}^{T},\\ a_{1}=\left[A_{1}^{H}\left(1,1\right),A_{1}^{H}\left(2,2\right),...,A_{1}^{H}\left(n,n\right)\right], \end{array}$$

Equation (25) is obtained via the matrix property (see Equation (1.10.6) in [34]), Equation (26) holds since $q^{*}=s$. Note that Equation (26) has just same equation with Equation (7). Therefore, by applying Algorithm 3, the solution of Q can also be obtained given fixed R. Hence, the AO algorithm can be applied to multi-antenna PR case.

## 4. Simulation Results

To validate the performance and convergence of the proposed AO algorithm, various simulation results are provided in this section. We set $\mu_{l}=0$ and $\mu_{u}=0$, $u_{l}=0$ and $u_{u}=1$ for bisection algorithms, and the target accuracy is set as $\epsilon_{1}=\epsilon_{2}=\epsilon_{3}=\epsilon_{4}=10^{-4}$ in the absence of special instructions. All the channels were randomly generated as complex zero-mean Gaussian random variables with unit covariance.

### 4.1. Secrecy Rate and the Activeness of Constraints

In this subsection, we compute the secrecy rate of IRS assisted CR MISO WTC via the proposed AO algorithm, and compare its performance with several existing methods without IRS. Moreover, the interplay between TPC and IPC is also analyzed under both IRS-assisted and no IRS case.

**Figure 2:**
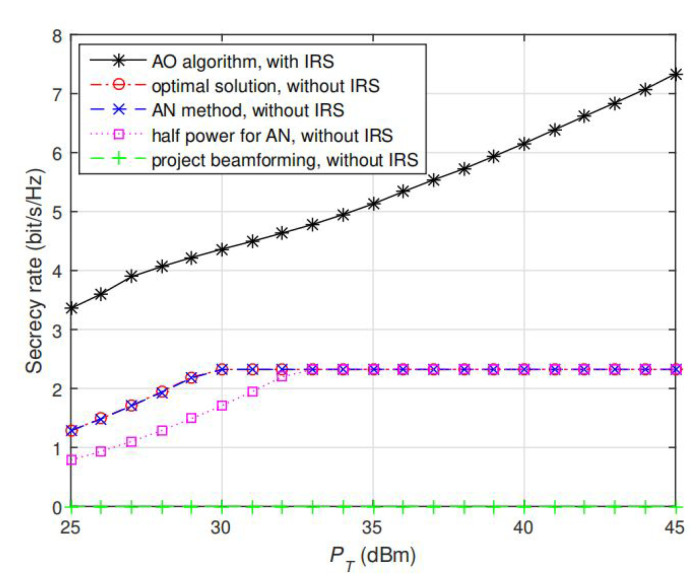
Secrecy rate performance comparison between AO algorithms with IRS and other solutions without IRS. $P_{T}$ is fixed at 30 dBm, the channels are randomly generated. Our proposed algorithm significantly boosts the secrecy rate compared with the existing solutions without IRS.

Firstly, we randomly generate the channels as

$$\begin{array}{cc} h_{AB}= & [0.64-0.79j,0.18-0.51j,1.34-1.24j],\\ h_{AE}= & [0.00-0.37j,0.70-0.21j,-0.34-0.11j],\\ h_{IB}= & [1.38-0.43j,-0.33+0.50j,-0.46+0.20j],\\ h_{IE}= & [-1.04-0.36j,-0.49-0.55j,-0.61+0.30j],\\ h_{IP}= & [0.72+0.54j,-0.18+0.09j,-0.39-0.19j], \end{array}$$

$$\begin{array}{cc} h_{AI}= & \left[\begin{array}{ccc} -0.15-0.93j & 0.30-0.88j & 0.62+0.16j\\ 1.51+0.92j & 0.08-0.13j & -1.57+1.49j\\ -0.46+0.70j & -0.76-0.53j & -1.38-0.62j \end{array}\right] \end{array}$$

so that the eigenvalues of $h_{AB}^{H}h_{AB}-h_{AE}^{H}h_{AE}$ are {4.60,0,−0.75}, i.e., the channel of direct link is non-degraded (since there exists negative eigenvalue –0.75). Particularly, we consider a worst case by setting $h_{AP}=0.5h_{AB}$, i.e., both PR and Bob are located in the same direction so that the channels $h_{AP}$ and $h_{AB}$ are strongly correlated and hence as $P_{T}$ is increasing, the secrecy rate is likely to saturate with transmit power under no IRS case (since PR is easily to receive the interference in this case so that IPC is becoming active and full power cannot be used for signaling). Then, we compare the performance of proposed AO algorithm with several methods without IRS: optimal solutions in [6], sub-optimal AN-aided solutions. We note that although the proposed model in [13] is a simultaneous information and wireless transfer based CR MIMO WTC, its proposed AN design and algorithm also applies for regular no IRS CR MISO WTC. In [13], sub-optimal project beamforming solutions by projecting the interference to the null space of matrix $h_{AP}^{H}h_{AP}$ as well as another AN-aided sub-optimal solutions by putting half of the total power for signaling via the solution in [6] and the residual half of the power for generating AN to the null space of both $h_{AB}^{H}h_{AB}$ and $h_{AP}^{H}h_{AP}$ (so that only Eve could receive AN signals). According to the results illustrated in Figure 2, note that our proposed IRS-assisted design and algorithm achieves significantly better secrecy rate performance than all the other solutions under no-IRS assisted case. The reason is that via optimizing the phase shift coefficient for the reflecting elements, the reflected signal by the IRS and the direct non-reflected signal can be constructively added at Bob but destructively added at Eve, thus providing new degree of freedom. Also note that all the secrecy rates returned by the optimal solutions and two sub-optimal AN methods without IRS saturates as $P_{T}$ is increasing, since TPC is inactive and IPC is active so that Alice cannot use more power for signal transmission; however, for the IRS-assisted case, the secrecy rate is still keep increasing with $P_{T}$ as if there is no IPC at all. The reason is that the reflected signal by the IRS and the direct non-reflected signal also destructively added to the PR. Therefore, although PR and Bob are in the same direction, Alice can still apply more power for the transmission through the Alice–IRS–Bob link and the QoS at PR is also not affected. In addition, the project beamforming solution has zero secrecy rate performance since the signals are completely nulled out at Bob and hence secrecy communication is not achievable.

**Figure 3:**
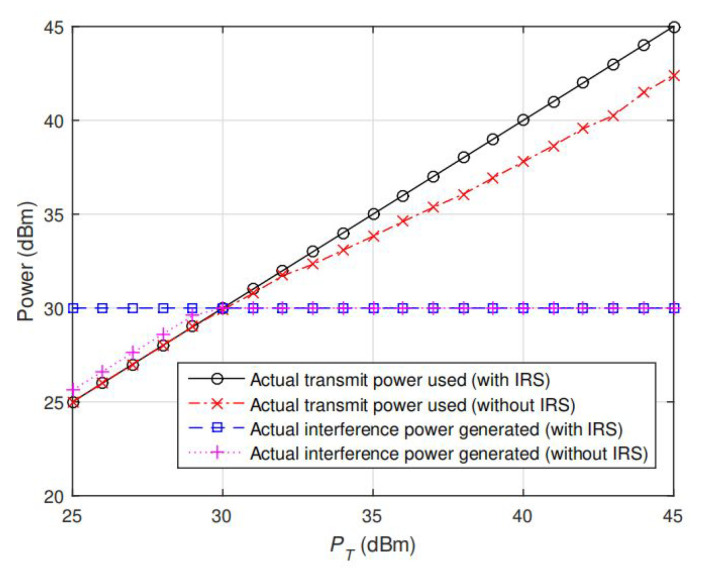
The actual transmit power used at Alice and actual interference power generated to PR under IRS-assisted and no-IRS case under same settings as in Figure 2. Note that $tr\left(R\right)=P_{T}$ always holds under IRS-assisted case, i.e., total power constraint (TPC) is always active even when IPC is active.

To see how the interplay between TPC and IPC is returned by the proposed AO algorithm, Figure 2 and Figure 3 show the corresponding secrecy rate and the actual power consumption at Alice (i.e., tr(R)). Note that in Figure 2, the secrecy rate gradually saturates with $P_{T}$ for no IRS design, since PR has better quality of channel than Bob. Therefore, as $P_{T}$ is increasing, full power allocation is not the optimal signaling strategy, or the interference generated to PR will exceed the threshold $P_{T}$. However, for the IRS-assisted design, the secrecy rate keeps increasing with $P_{T}$ as if the IPC is not present at all. The main reason is that IRS helps to make the signals from Alice–PR link and Alice–IRS–PR link destructively added at PR so as to eliminate the interference and hence full power allocation is always optimal at Alice. This can be further validated in Figure 3, in which the actual power used at Alice (i.e., tr(R)) and actual interference power generated to PR (i.e., $h_{P}Rh_{P}^{H}$) returned by AO algorithm under the same setting of channels and $P_{T}$ as in Figure 2 ($P_{T}=30dBm$), and the results are also compared with the optimal solution without IRS. From Figure 3, we note that the actual total power (tr(R) returned by AO algorithm) of the IRS case is always equal to $P_{T}$ and the $h_{P}Rh_{P}^{H}=P_{I}$, i.e., the actual total power ( tr(R) returned by AO algorithm) of IRS case is still linearly increasing with $P_{T}$. This means Alice can always use full power for transmitting, and the interference generated to PR still does not exceed the threshold $P_{I}$, even when the IPC is active, which makes the secrecy rate increase with $P_{T}$ illustrated in Figure 2. The main reason is that IRS helps to make the signals from Alice–PR link and Alice–IRS–PR link destructively added at PR so as to eliminate the interference and hence full power allocation is always optimal at Alice.

Otherwise, From Figure 3, we can find that the actual total power ( tr(R) returned by AO algorithm) of no IRS case is equal to $P_{T}$ and the $h_{P}Rh_{P}^{H}<P_{I}$, when $P_{T}\leq 30$, i.e., IPC is inactive so that Alice can use full power for transmitting, and the interference generated to PR still does not exceed the threshold $P_{I}$. However, when $P_{T}>30$, we note that the $tr\left(R\right)<P_{T}$ (although tr(R) is still growing with $P_{T}$ ) and $h_{P}Rh_{P}^{H}=P_{I}$ appears, i.e., Alice can not full power for transmitting due to IPC active. This is because if it signals at full power, the interference power to PR will exceed the threshold ($P_{I}=30$) and affect the communication quality of PR, which is contrary to the IPC and TPC of the CR system. Hence, from these results we find that IRS not only greatly helps enhance the secrecy rate, but also eliminates the restriction brought by IPC on secrecy performance under some certain channel conditions, which bring broad prospects for future development of CR systems.

In fact, the sufficient and necessary condition for the secrecy rate to grow unbounded with $P_{T}$ given fixed $P_{I}$ is that the effective channel $h_{i}=h_{Ai}+h_{Ii}QH_{AI}$, i∈{B,E,P} satisfy [10]

(27) $$\begin{array}{c} N\left(h_{E}^{H}h_{E}\right)\cap N\left(h_{P}^{H}h_{P}\right)\notin N\left(h_{B}^{H}h_{B}\right). \end{array}$$

Without IRS (i.e., Q=0) and if $h_{AP}=0.5h_{AB}$, then $N\left(h_{P}^{H}h_{P}\right)=N\left(h_{AP}^{H}h_{AP}\right)=N\left(h_{B}^{H}h_{B}\right)=N\left(h_{AB}^{H}h_{AB}\right)$ so that (31) does not hold (even when $N\left(h_{AP}^{H}h_{AP}\right)\cap N\left(h_{AE}^{H}h_{AE}\right)=\emptyset$). With IRS, Q can be properly optimized by our AO algorithm so as to meet (31), which is the key reason why full power allocation is always optimal and the secrecy rate is keep increasing with $P_{T}$.

**Figure 4:**
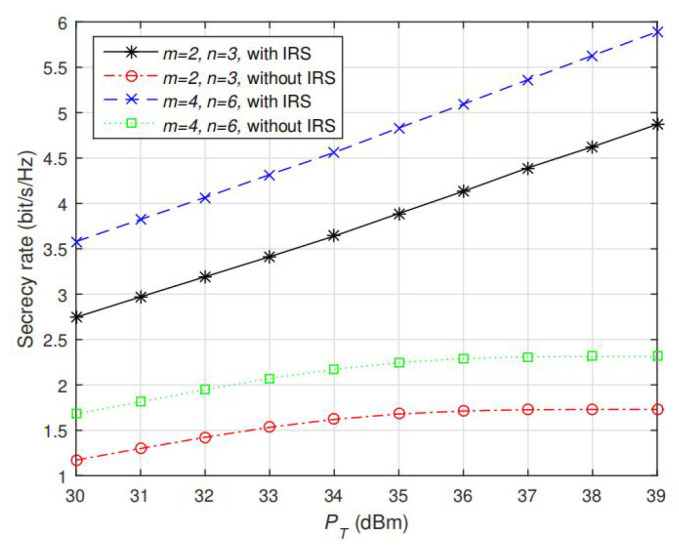
Average secrecy rate of intelligent reflecting surface (IRS)-assisted case using AO algorithm and no-IRS case using optimal solution in [6] under different settings of m=2,4 and n=3,6. $P_{T}$ is fixed at 30 dBm.

**Figure 5:**
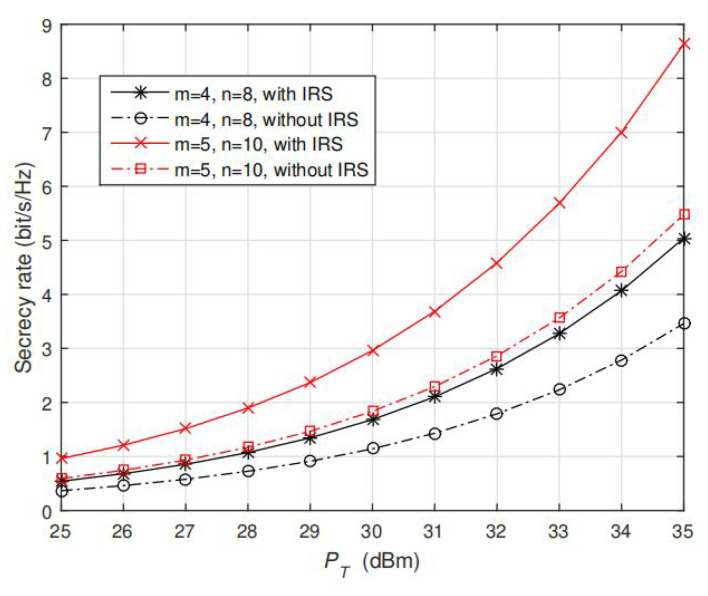
Average secrecy rate of IRS-assisted case using AO algorithm and no-IRS case using optimal solution in [6] under different settings of m=4,5 and n=8,10. $P_{T}$ is fixed at 30 dBm.

To validate the performance of AO algorithm under more channel realizations, we keep the setting $h_{AP}=0.5h_{AB}$, and compute the average secrecy rate under 100 randomly generated channels. The results are shown in Figure 4 and Figure 5, note that our proposed solution still achieves significantly better performance than the optimal solutions without IRS given different values of *m* and *n*. With the aid of IRS, the average secrecy rate keeps (almost linearly) increasing with $P_{T}$, which is significantly different from no IRS case (that the saturation appears as IPC is active). The reason is that via jointly optimizing the phase shift coefficient for the reflecting elements, the reflected signals and the direct non-reflected signal can be constructively added at Bob but destructively added at Eve so that the secrecy performance can be enhanced. Figure 5 also shows that as m and n increase, a larger degree of freedom can be obtained so that a larger secrecy rate can be achieved. Collectively, according to the aforementioned results in Figure 2, Figure 3 and Figure 4, we conclude that our proposed AO algorithm based on IRS-assisted design greatly enhances the secondary user’s secrecy performance in CR network.

### 4.2. Convergence of the Proposed AO Algorithm

**Figure 6:**
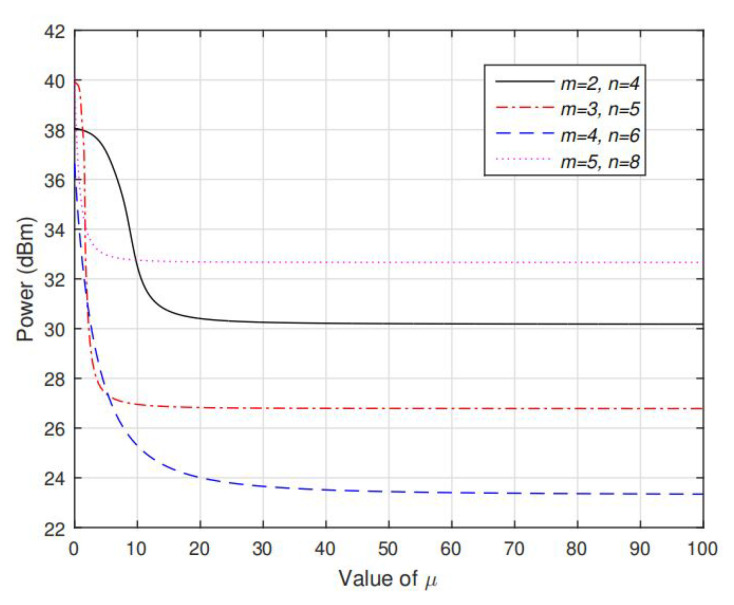
Value of $\tilde{g}\left(s\left(\mu \right)/\tilde{s}\right)$ as function of μ under different settings of *m*, *n*. $\tilde{s}$ is set as ${\left[1,1,...,1_{n}\right]}^{T}$, given each fixed value of μ, s(μ) is set as Equation (22) under the inactive Equation (13). Note that $\tilde{g}\left(s\left(\mu \right)/\tilde{s}\right)$ is monotonically decreasing in μ.

**Figure 7:**
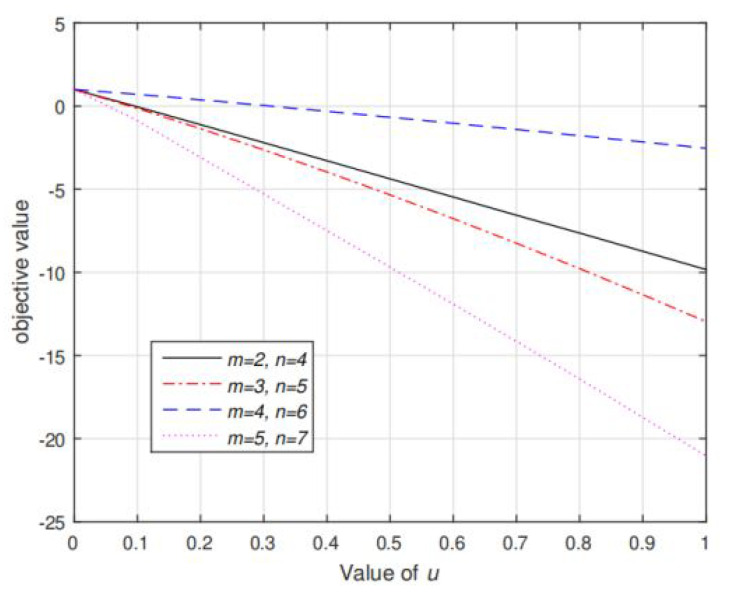
Value of the objective function f(s/u) as function of *u* under different settings of *m*, *n*. Given each fixed value of *u*, s/u is computed via Algorithm 2. Note that f(s/u) is monotonically decreasing in *u*.

**Figure 8:**
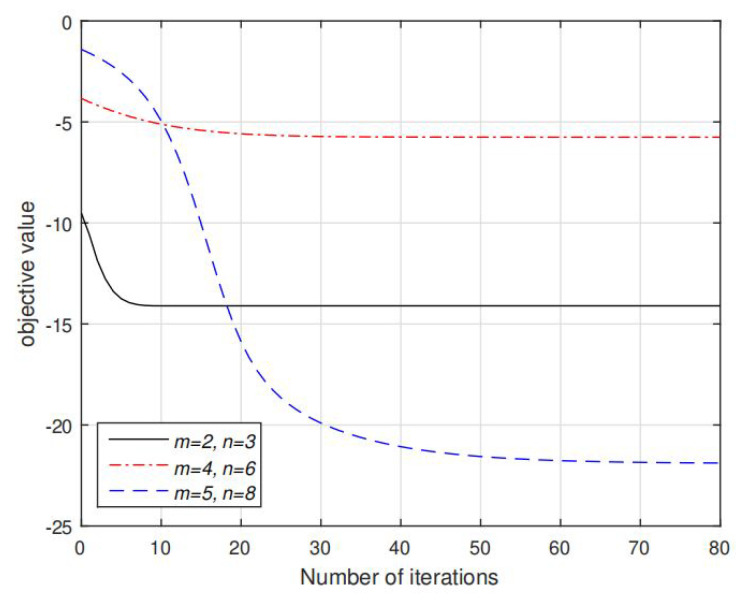
Convergence of $f_{k}$ in Algorithm 2 as function of iteration *k* under different settings of *m*, *n*. μ is fixed with 0.5.

In this subsection, we validate the convergence of proposed AO algorithm. In Figure 5, Figure 6, Figure 7 and Figure 8, the results are all computed under fixed $P_{T}=P_{I}=35dBm$, and randomly generated channels.

Figure 6 and Figure 7 shows the value of function $\tilde{g}\left(s\left(\mu \right)/\tilde{s}\right)$ and f(s/μ) respectively. It can be seen that under various settings of *m* and *n*, $\tilde{g}\left(s\left(\mu \right)/\tilde{s}\right)$ is strictly decreasing in μ and f(s/u) is strictly decreasing in *u*, which validates the Propositions 2 and 5. Note that in Figure 6, $\tilde{g}\left(s\left(\mu \right)/\tilde{s}\right)$ is firstly quickly decreasing when 0<μ<20, and then decreasing slowly until converge when μ>20. This means that s is very sensitive to low value of μ and as μ is increasing to large enough, μ has very little effect on the value of *s*. Based on our extensive simulations, the target μ such that $\tilde{g}\left(s\left(\mu \right)/\tilde{s}\right)={\tilde{P}}_{I}$ always exists at the range where $\tilde{g}\left(s\left(\mu \right)/\tilde{s}\right)$ is quickly decreasing. In addition, in Figure 6, the optimal *u* such that f(s/u)=0 must lies in (0, 1). If u>1, then the corresponding transmission rate at Alice is less than that in Eve and hence secrecy communication cannot be realized.

**Figure 9:**
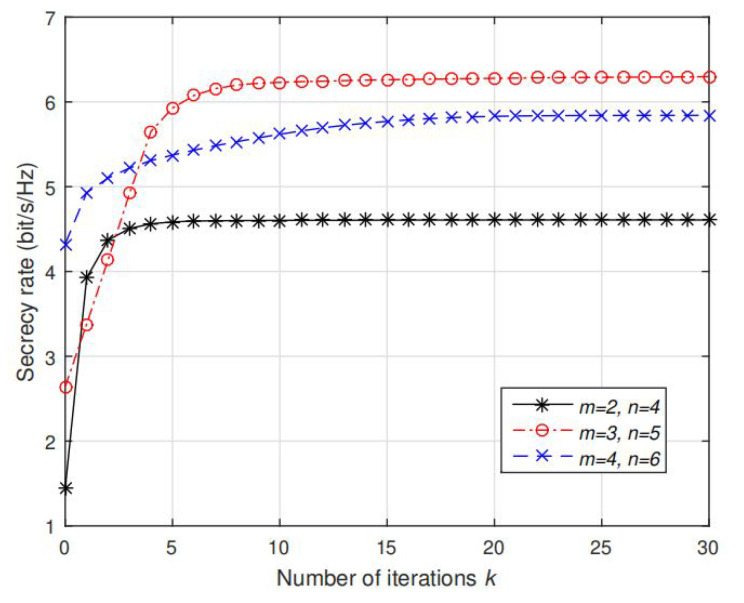
Convergence of $C_{s}\left(R_{k},K_{k}\right)$ in Algorithm 4 as function of iteration *k* under different settings of *m*, *n*.

Finally, Figure 8 and Figure 9 illustrate the convergence of the MM algorithm to solve P6 and the proposed AO algorithm for solving P1 under different settings of *m* and *n*, respectively. We plot the objective value $f_{k}$ in Algorithm 2 and $C_{s}\left(R_{k},Q_{k}\right)$ in Algorithm 4 versus the number of iterations *k* under several randomly generated channels with different settings of *m* and *n*. Note that for all considered *m* and *n*, $f_{k}$ is monotonically decreasing function and $C_{s}\left(R_{k},Q_{k}\right)$ is monotonically increasing function, which validate the convergence of MM Algorithm 2 and AO Algorithm 4. It requires about 5 to 50 iterations for $f_{k}$ to converge, and 5 to 19 iterations are required to converge for $C_{s}\left(R_{k},Q_{k}\right)$. Furthermore, note that larger settings of *m* and *n* requires more iterations to converge to the same accuracy. The reason is that the dimensions of variable R and Q become larger so that the AO algorithm requires more iterations to optimize each element in these variables. In addition to these results, our other extensive simulations also indicate that a monotonic convergence of AO algorithm is guaranteed.

## 5. Conclusions

In this paper, an IRS-assisted CR Gaussian MISO wiretap channel is studied. To maximize the secrecy rate of secondary user in this channel subject to TPC at transmitter as well as IPC at PR, an AO algorithm is proposed in which the transmit covariance for transmitter and phase shift coefficient for IRS are optimized alternatively by fixing the other as constant. Simulation results have validated the performance and convergence of the proposed AO algorithm. It is shown that with IRS, the secrecy rate of secondary user can be greatly enhanced compared with existing solutions for no IRS case. And as IPC is active, full power allocation is still optimal so that the secrecy rate keeps increasing with transmit power, which is significantly different from no IRS case. 

## Appendix A

### Appendix A.1. Proof of Proposition 1

Proposition 1 can be obtained by following the step of proof for Proposition 1 and 2 in [10]. Let $W_{j}=h_{j}^{H}h_{j}$, j∈{B,E,P} then the stationary condition in the KKT condition of P2 can be expressed as

$$\begin{array}{c} {\left(I+W_{B}R\right)}^{-1}W_{B}-W_{E}{\left(I+RW_{E}\right)}^{-1}+M=\alpha_{1}I+\alpha_{2}W_{P} \end{array}$$

where $\alpha_{1}$, $\alpha_{2}$, and M represent the Lagrange multipliers corresponding to TPC, IPC, and R≥0 in P2 respectively. Using the same steps of proof in [10] by manipulating the above stationary condition, one obtains that the optimal signaling strategy for Alice is over the positive directions of the difference channel $W_{B}-W_{E}$, i.e.,

(A1) $$\begin{array}{c} x^{H}\left(W_{B}-W_{E}\right)w\geq 0 \end{array}$$

for any information signal $x\in R(R_{opt})$, and signaling over negative directions of $W_{B}-W_{E}$ would provide more information to Eve so that secrecy communication can not be guaranteed. Hence, according to Proposition 2 in [10],

(A2) $$\begin{array}{c} rank\left(R_{opt}\right)=min\left(m,1\right)=1 \end{array}$$

which completes the proof.

### Appendix A.2. Proof of Proposition 2

Consider $\mu_{1}>\mu_{2}>0$, and let $s(\mu_{1})$, $s(\mu_{2})$ denote the solution of P7 with $\mu_{1}$, $\mu_{2}$. Assume $s(\mu_{1})$ is the global optimal solution of P7, then

(A3) $$\begin{array}{c} L\left(s\left(\mu_{1}\right),\mu_{1},\upsilon_{i}\right)\leq L\left(s\left(\mu_{2}\right),\mu_{1},\upsilon_{i}\right). \end{array}$$

Similarly, assume $s(\mu_{2})$ is the global optimal solution of P7, then

(A4) $$\begin{array}{c} L\left(s\left(\mu_{2}\right),\mu_{2},\upsilon_{i}\right)\leq L\left(s\left(\mu_{1}\right),\mu_{2},\upsilon_{i}\right). \end{array}$$

Combining these two inequalities, one obtains

(A5) $$\begin{array}{c} \left(\mu_{1}-\mu_{2}\right)\tilde{g}\left(s\left(\mu_{1}\right)/\tilde{s}\right)\leq \left(\mu_{1}-\mu_{2}\right)\tilde{g}\left(s\left(\mu_{2}\right)/\tilde{s}\right). \end{array}$$

Since $\mu_{1}-\mu_{2}$, then $\tilde{g}\left(s\left(\mu_{1}\right)/\tilde{s}\right)\leq \tilde{g}(s\left(\mu_{2}\right)/\tilde{s}$, from which the proposition holds.

### Appendix A.3. Proof of Proposition 3

Given initial point $\tilde{s}$, consider the global optimal solution for P7 is $\tilde{s}$, then the following holds

(A6) $$\begin{array}{c} \tilde{g}\left(\hat{s}/\tilde{s}\right)={\tilde{P}}_{I}. \end{array}$$

Then, consider the optimal μ returned by the Algorithm 1 is $\hat{\mu }$, and the corresponding s as $s(\hat{\mu })$, it can be known that $\hat{\mu }$ returned by bisection search makes the following holds

(A7) $$\begin{array}{c} \tilde{g}\left(s\left(\hat{\mu }\right)/\tilde{s}\right)={\tilde{P}}_{I}. \end{array}$$

Now we assume that $s(\hat{\mu })$ is not the global optimal solution of P7, then

(A8) $$\begin{array}{c} -\tilde{f}\left(s\left(\hat{\mu }\right)/\left(u,\tilde{s}\right)\right)\leq -\tilde{f}\left(\hat{s}/\left(u,\tilde{s}\right)\right). \end{array}$$

Note that $s(\hat{\mu })$ is the global optimal solution of P9 under $\mu =\hat{\mu }$, then the following holds

(A9) $$\begin{array}{c} -\tilde{f}\left(s\left(\hat{\mu }\right)/\left(u,\tilde{s}\right)\right)-\mu \tilde{g}\left(s\left(\hat{\mu }\right)/\tilde{s}\right)\geq -\tilde{f}\left(\hat{s}/\left(u,\tilde{s}\right)\right)-\mu \tilde{g}\left(\hat{s}/\tilde{s}\right). \end{array}$$

By substituting Equations (A6) and (A7) into Equation (A9) and after some manipulations, one obtains

(A10) $$\begin{array}{c} -\tilde{f}\left(s\left(\hat{\mu }\right)/\left(u,\tilde{s}\right)\right)\geq -\tilde{f}\left(\hat{s}/\left(u,\tilde{s}\right)\right). \end{array}$$

Combining Equations (A8) and (A10),

(A11) $$\begin{array}{c} -\tilde{f}\left(s\left(\hat{\mu }\right)/\left(u,\tilde{s}\right)\right)=-\tilde{f}\left(\hat{s}/\left(u,\tilde{s}\right)\right). \end{array}$$

holds so that $s\left(\hat{\mu }\right)=\hat{s}$, i.e., $s(\hat{\mu })$ returned by Algorithm 1 is the global optimal solution of P7, from which the proof is complete.

### Appendix A.4. Proof of Proposition 4

Firstly, it can be known that $s_{k}$ always satisfies the unit modulus constraint of Equation (4) for each iteration *k*. Secondly, Since $c_{3}+\tilde{g}\left(s/\tilde{s}\right)$ is an surrogate function of g(s), then given $s_{k}$, one obtains

(A12) $$\begin{array}{c} g\left(s_{k}\right)=c_{3}+\tilde{g}\left(s_{k}/s_{k}\right)\leq \tilde{g}\left(s_{k+1}/s_{k}\right)\leq P_{I}. \end{array}$$

where the first inequality holds due to the property of surrogate function and where the second inequality holds since $s_{k+1}$ is also a feasible solution of P7. Hence, the IPC $g\left(s_{k}\right)\leq P_{I}$ is also guaranteed during each iteration.

Thirdly, the sequence $\left\{f\left(s_{k}/u\right)\right\}$ is guaranteed to converge due to the property of MM algorithm:

$$\begin{array}{c} f\left(s_{k}/u\right)=\tilde{f}\left(s_{k}/\left(u,s_{k}\right)\right)\geq \tilde{f}\left(s_{k+1}/\left(u,s_{k}\right)\right)\geq \tilde{f}\left(s_{k+1}/\left(u,s_{k+1}\right)\right)=f\left(s_{k+1}/u\right). \end{array}$$

Therefore, $f\left(s_{k}/u\right)\geq f\left(s_{k+1}/u\right)$ so that $f(s_{k}/u)$ is a monotonically decreasing sequence with *k*.

In the following, we show that the results produced by Algorithm 2 is a KKT point of P6. Consider the converged point by Algorithm 2 is $\hat{s}$, which is also the global optimal solution of P7 given initial point $\tilde{s}=\hat{s}$, then the corresponding KKT conditions of P7 are

(A13) $$\begin{array}{c} \nabla_{\hat{s}}\tilde{f}\left(s/\left(u,\hat{s}\right)\right){|}_{s=\hat{s}}+\mu \nabla_{\hat{s}}\tilde{g}\left(s/\hat{s}\right){|}_{s=\hat{s}}+\sum_{i=1}^{n}\upsilon_{i}\nabla_{\hat{s}}\left|q_{i}\right|=0, \end{array}$$

(A14) $$\begin{array}{c} \mu \left(\tilde{g}\left(\hat{s}/\hat{s}\right)-{\hat{P}}_{I}\right)=0,\upsilon_{i}\left(\right|q_{i}\left|-1\right)=0,\mu \geq 0,\upsilon_{i}\geq 0 \end{array}$$

(A15) $$\begin{array}{c} \tilde{g}\left(\hat{s}/\hat{s}\right)\leq {\hat{P}}_{I},\left|q_{i}\right|=1, \end{array}$$

where ${\hat{P}}_{I}=P_{I}-{\bar{h}}_{AP}+2n\lambda_{1}\left({\bar{h}}_{AP}\right)-{\hat{s}}^{H}{\bar{H}}_{IP}\hat{s}$. Note that

(A16) $$\begin{array}{c} \nabla_{\hat{s}}\tilde{g}\left(s/\hat{s}\right){{|}_{s=\hat{s}}=\nabla_{\hat{s}}g\left(s\right)|}_{s=\hat{s}},\tilde{g}\left(\hat{s}/\hat{s}\right)=g\left(\hat{s}\right), \end{array}$$

by substituting Equation (A16) into Equations (A13)–(A15), we find that this is just the KKT conditions of P6. Therefore, the converged point $\hat{s}$ is a KKT point of P6, from which the proof is complete.

### Appendix A.5. Proof of Proposition 5

Consider $0<u_{1}<u_{2}$, then according to the property of surrogate function in the MM algorithm, we have

(A17) $$\begin{array}{c} f\left(s\left(u_{2}\right)/u_{2}\right)\leq \tilde{f}(s\left(u_{2}\right)/\left(u_{2},s\left(u_{1}\right)\right)\leq \tilde{f}(s\left(u_{2}\right)/\left(u_{2},s\left(u_{1}\right)\right)=f\left(s\left(u_{1}\right)/u_{2}\right)\\ \leq f(s\left(u_{1}\right)/u_{1}) \end{array}$$

where Equation (A17) holds since $u_{1}<u_{2}$. Therefore, $f\left(s\left(u_{2}\right)/u_{2}\right)\leq f\left(s\left(u_{1}\right)/u_{1}\right)$ and hence f(s(u)/u) is a monotonically decreasing function in *u*.
